# Investigation of Mechanical Properties of Large Shape Memory Alloy Bars under Different Heat Treatments

**DOI:** 10.3390/ma13173729

**Published:** 2020-08-24

**Authors:** Liping Kang, Hui Qian, Yuancheng Guo, Chenyang Ye, Zongao Li

**Affiliations:** School of Civil Engineering, Zhengzhou University, Zhengzhou 450001, China; kangliping0720@gmail.com (L.K.); guoyc@zzu.edu.cn (Y.G.); yechenyang@gs.zzu.edu.cn (C.Y.); lizongao@gs.zzu.edu.cn (Z.L.)

**Keywords:** heat treatment, mechanical property, self-centering, shape memory alloy, superelasticity

## Abstract

Shape memory alloys (SMAs) are a class of functional materials that possess unique thermomechanical properties, such as shape memory effect (SME), superelasticity (SE), damping, and good fatigue and corrosion resistance, which enable them to become ideal materials for applications in earthquake engineering. Numerous studies have shown that the mechanical properties of superelastic SMAs mainly depend on the wire form, or the relationship between the microstructure and thermally induced phase transitions. However, extremely few studies have elucidated the effects of the heat-treatment strategy, size effect of large diameters, and cyclic loading. Herein, the mechanical properties of SMA bars, such as residual strain, energy dissipation, and equivalent damping ratio, were studied with different heat-treatment strategies, cyclic loadings, and strain amplitudes; this was achieved by conducting cyclic tensile tests on SMA bars with four different diameters. The results indicate that the maximum phase transformation stress was obtained with a 14 mm SMA bar subjected to heat treatment at 400 ℃ for 15 min. The mechanical properties were relatively stable after five loading–unloading cycles, which should be considered in engineering applications. The test results provide a mechanical basis for using large SMA bars in self-centering structures in seismic regions.

## 1. Introduction

Shape memory alloys (SMAs) have drawn significant attention owing to their remarkable shape memory effect and superelasticity. SMAs have two main phases, namely, the cubic crystal structure (B2) called austenite (A) and the monoclinic structure (B19’) called martensite (M) [1]. The shape memory effect occurs when the martensitic phase of the SMA is deformed; however, the residual deformation can be recovered by heating. The SMA shows superelasticity when its austenitic state is deformed under external loading, and the deformation can be recovered spontaneously upon unloading. As intelligent materials, SMAs are widely used in various cutting-edge technology fields, such as aerospace, robotics, and medicine [2].

Over the last decade, increasing research and new applications have been reported in civil engineering for achieving material cost reduction, production process improvements, and a clearer understanding of the mechanical properties of SMAs, especially those of NiTi SMAs. Various kinds of SMA-based vibration control devices, such as isolators [3,4,5,6,7] and dampers [8,9,10,11], have been developed by exploiting the energy dissipation capacity and recentering ability of SMAs. Development in the machining and connection technology has made it possible to use superelastic SMA bars as reinforcement materials in the construction industry, such as in beams [12,13,14], columns [15,16], beam–column joints [17,18], and shear walls [19,20]. SMA wires are often applied to confine the concrete cylinder [21] or retrofit the reinforced concrete column [22] for seismic application based on their unique mechanical behavior. The recovery stress generated by unloading in stress-induced phase transformation is particularly important in these applications.

The chemical composition and heat treatment exert a significant effect on the transformation temperature and stress of SMAs [23,24,25]. The mechanical properties of SMAs are extremely sensitive to the thermomechanical heat treatment. Yoon and Yeo [26] discussed the effect of the time and temperature of the heat treatment on the phase transformation temperature and thermomechanical properties of 54.5Ni-45.5Ti (wt.%) 1 mm diameter SMA wires. They reported that the heat-treatment time should be longer than 15 min in order to obtain a stable phase transformation. Furthermore, the samples tended to lose hyperelasticity once the heat-treatment temperature exceeded 500 ℃. Eggeler et al. [27] presented the effect of 450 ℃ aging on the transformation behavior of SMAs using differential scanning calorimetry (DSC). They showed that the transformation temperature increased as the aging time increased. Kaya et al. [28] suggested that the transformation temperatures of NiTi alloy could be tailored by aging. An attempt was made to study the effect of heat treatment on the recovery stress of the 55Ni-45Ti (wt.%) alloy and the transformation temperatures. The maximum recovery stress significantly decreased when the annealing temperature was above 600 ℃. Therefore, SMAs should be heat-treated at no more than 450 ℃ [29]. However, Sadrnezhaad [30] proposed that above 600 ℃, the parent-phase yield stress increased for 57.06Ni-42.94Ti (wt.%); therefore, its optimum heat-treatment temperature was approximately 600 ℃. DesRoches et al. [31] conducted a comparative evaluation of the cyclic properties of SMA wires and bars and showed that nearly ideal superelastic properties could be achieved in both the wire and the bar. Hence, SMA bars can be used in seismic applications. Lin et al. [32] investigated the fatigue properties of the SMA and pointed out that the fatigue life became shorted and the energy consumption decreased with the increase of temperature. Balagna et al. [33] and Mentz et al. [34] developed a series of experimental programs which proved that the precipitation of the Ni_4_Ti_3_ phase influenced the transformation and mechanical behavior of the SMA. Wang et al. [35] conducted a series of mechanical tests on large SMA bars by considering the size effect and different heat-treatment strategies. They showed that the heat-treatment strategies needed to be adjusted for bars with different diameters in order to obtain the optimal mechanical response. The annealing process could decrease the transformation temperature required to obtain superelasticity at room temperature. When the austenite finish temperature *A_f_* is lower than the room temperature, SMA exhibits superelasticity under an external force. The ambient temperature should preferably be between *A_f_* and *A_f_* + 40 ℃ [36]; however, *A_f_* is usually higher than room temperature. Therefore, *A_f_* should be reduced by carrying out different heat treatments in order to achieve the desirable superelasticity for SMA wires.

A review of previous works showed that significant efforts have been made to focus on the crystallography and mechanics at a microstructural level from the materials science point of view. However, very few studies have been conducted on the unique performance of bars with large diameters, which are generally required for civil engineering applications. In particular, not much attention has been paid to properties such as self-centering ability, transformation stress, fatigue property, and energy dissipation, which are specific requirements for the structural application of SMAs. Four diameters of bars were selected, which were used as reinforcement in beams, joints, and shear walls in structural engineering tests. It is necessary to precisely understand and quantify their mechanical properties in order to develop SMA-reinforced structural members with better seismic performance. This study aims to experimentally investigate the effects of the heat-treatment strategy, cyclic loading, and unequal cyclic loading on the mechanical properties of SMA bars.

## 2. Materials and Methods

**Figure 1:**
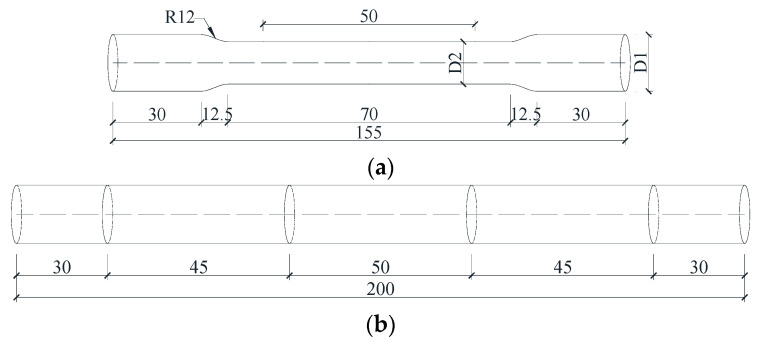
Dimensions of the test specimens: (**a**) 10 mm and 14 mm, (**b**) 5.5 mm and 8 mm.

**Figure 2:**
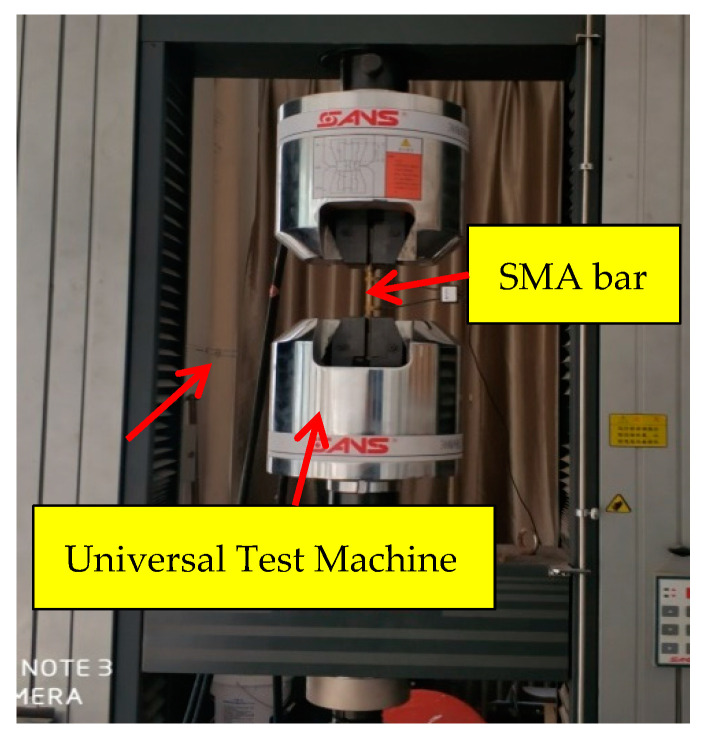
Experimental setup for mechanical tests.

The SMA bars were made of commercial Ti-55.8 wt.% Ni by the Baoji Seabird Metal Material Co., Ltd. (Baoji, China). A wide variety of sizes were selected to meet the needs of different applications, such as beams, joints, and shear walls. The diameters of the bars ranged from 5.5 to 14 mm. The reference diameter was 14 mm. According to the national standard for metallic tensile testing (GB/T 228.1-2010), the raw bars were machined into dog-bone shape coupons with a 14 mm or 10 mm diameter over the working segments. The size is shown in Figure 1a, and the original gauge length was 50 mm. The specimen with a diameter of 8 mm or 5.5 mm was not machined into a dog-bone shape, as illustrated in Figure 1b. The original gauge length was 50 mm and the parallel length was 140 mm, which was long enough to avoid stress concentration in the measurement section and met the requirements of the standard (GB/T 228.1-2010). For all the specimens, the mechanical tests were carried out using displacement control with a rate of 1 mm/min via a universal test machine (UTM) (CMT5205, MTS Industrial Systems(China) Co., Ltd., Shenzhen, China), as shown in Figure 2. Table 1 shows a summary of the specimens, heat treatments, and number of cycles. 

The bars were first heat-treated using an electric furnace (RJ2-60-9, Jiangsu Hengli Farnaces Co., Ltd, Jiangsu, China) at 400 ℃ or 450 ℃ for various durations (ranging from 15 to 30 min) and then water-quenched. The specific method of heat treatment was as follows: (i) The furnace temperature was increased to 400 ℃ or 450 ℃. (ii) The furnace temperature was maintained at the target temperature for 10 min to ensure temperature stability. (iii) The SMA bars were quickly placed inside the furnace. (iv) The bars were heat-treated for 15, 20, and 30 min at the target temperature. (v) After removal from the furnace, the SMA bars were water-quenched.

**Figure 3:**
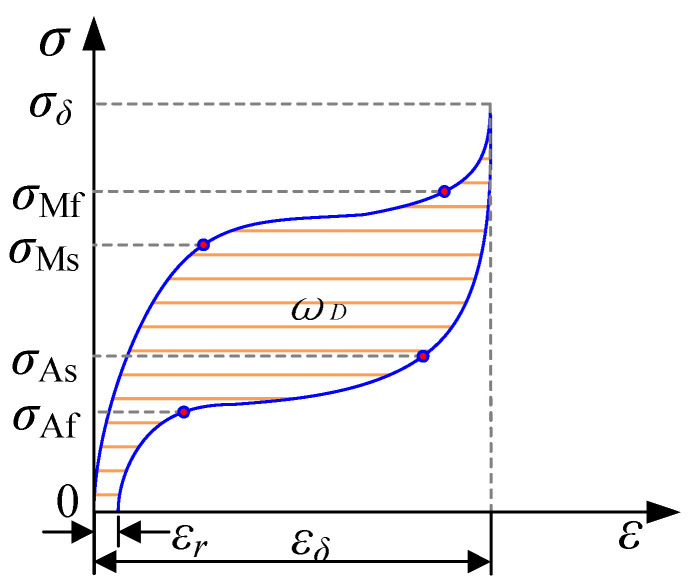
Schematic stress–strain curve and mechanical parameters of a superelastic shape memory alloy (SMA).

Figure 3 shows the stress–strain curve and mechanical parameters of a superelastic SMA. There are four phase transformation stresses: martensite start stress $\sigma_{Ms}$, martensite finish stress $\sigma_{Mf}$, austenite start stress $\sigma_{As}$, and austenite finish stress $\sigma_{Af}$. The following mechanical characteristic parameters were used to better analyze the changes in the mechanical properties of SMA bars under different working conditions:

(1) Residual strain $\varepsilon_{r}$: Residual strain refers to the remaining strain when the specimen returns to the state of zero stress.
(2) Energy consumption per cycle $\omega_{D}$: This is the area inside the hysteresis loop during each loading–unloading cycle.
(3) Secant stiffness $k_{s}$:
  (1) $$k_{s\;}=\;\frac{F_{max}-F_{min}}{\delta_{max}-\delta_{min}},$$
  where, *F_max(min)_* are the forces and *δ_max(min)_* are the displacements.
(4) Equivalent viscous damping ratio $\xi_{eq}$: This represents the damping performance of SMA bars and is calculated as
  (2) $$\xi_{eq}=\frac{\omega_{D}}{2\pi k_{s}\delta^{2}}.$$

## 3. Results and Discussion

### 3.1. Heat-Treatment Strategy Response

**Figure 4:**
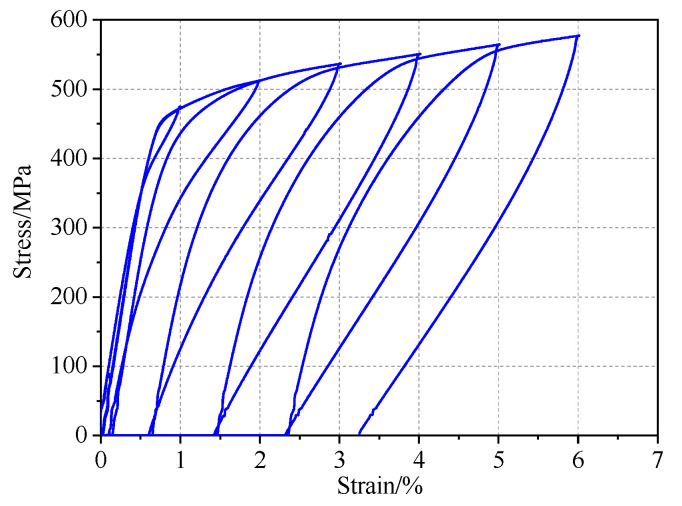
Hysteretic response with no heat-treated specimen.

**Figure 5:**
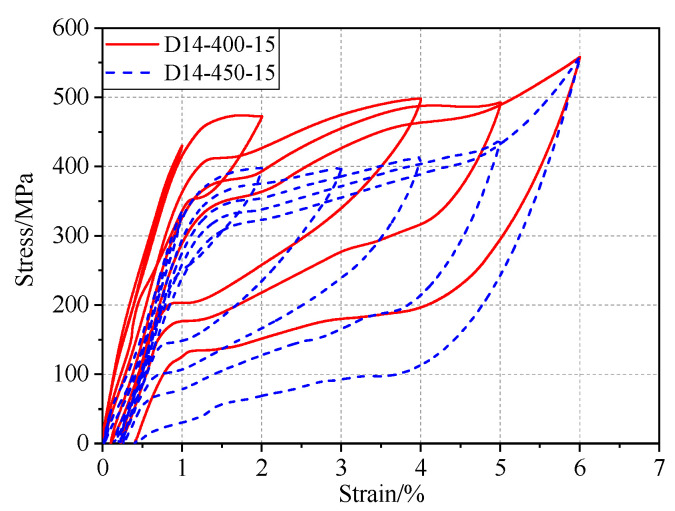
Hysteretic response under different temperatures. D14-400-15: 14 mm specimen heat-treated at 400 ℃ for 15 min; D14-450-15: 14 mm specimen heat-treated at 450 ℃ for 15 min.

The primary purpose of heat treatment is the development of the nickel-rich Ti_3_Ni_4_ precipitate, which is considered to assist the martensitic transformation and impede the occurrence of dislocation in the B2 matrix at a microscopic level. At the macroscopic level, it exhibits superelastic behavior and strain recovery. The specimen not subjected to heat treatment did not develop any superelasticity, and the stress–strain curve was ordinary (Figure 4). When the SMA bars received a moderate heat treatment, typical flag-shaped responses with satisfactory recentering capabilities were obtained (Figure 5). The label D14-400-15 in the figure indicates that the specimen with a diameter of 14 mm was heat-treated at 400 ℃ for 15 min. Evident degradations of the forward and reverse transformation plateaus were observed when the temperature was increased from 400 ℃ to 450 ℃. Young’s modulus also tended to decrease.

**Figure 6:**
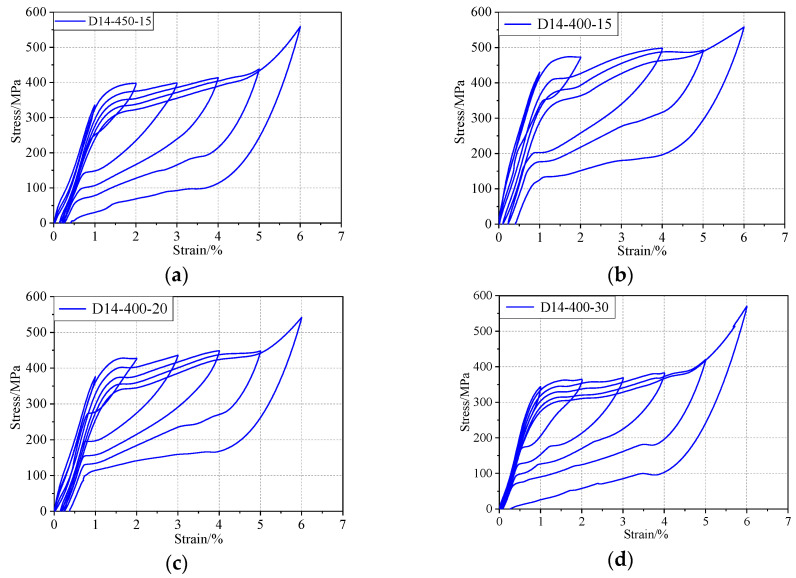
Hysteretic response of the specimens under different heat treatments: (**a**) 450 ℃ for 15 min, (**b**) 400 ℃ for 15 min, (**c**) 400 ℃ for 20 min, and (**d**) 400 ℃ for 30 min.

**Figure 7:**
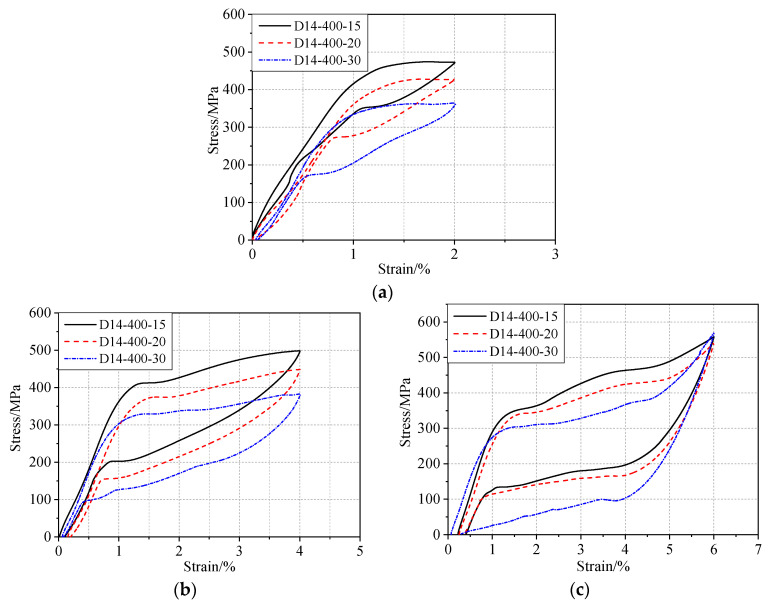
Comparison of SMA bars subjected to different heat-treatment durations: comparison of stress–strain diagrams at (**a**) 2% strain, (**b**) 4% strain, and (**c**) 6% strain.

Figure 6 presents recognizable flag-shaped responses with a residual strain less than 0.5% after experiencing the maximum strain of 6%, indicating a recovery rate of more than 90%. Figure 7 shows a comparison of the typical stress–strain diagrams at 2%, 4%, and 6% strain for different annealing durations. When the annealing temperature is constant, any increase in the annealing duration could lower the transformation plateau. The forward transformation stress (referred to as the loading plateau stress) and the recovery stress (referred to as the unloading plateau stress) decreased with increasing annealing duration (Figure 7). The austenitic end stress of the 15 min heat treatment was approximately 350 MPa, while that of the 30 min heat treatment reduced to approximately 170 MPa (Figure 7a). Therefore, the heat-treatment duration should be limited in order to obtain a higher recovery stress. 

Generally, further annealing could optimize the properties required for engineering applications. For the 14 mm bars, superior stress and recovery stress could be obtained by heat treatment at 400 ℃ for 15 min; the residual deformation was also small. The heat-treated temperature and duration may need to be adjusted for diverse bars in order to obtain the optimal mechanical response. More detailed heat-treatment rules for bars with different sizes and shapes of SMA specimens may be determined. In the subsequent analysis, all the test specimens were heat-treated at 400 ℃ for 15 min.

### 3.2. Cyclic Response at Constant Strain

**Figure 8:**
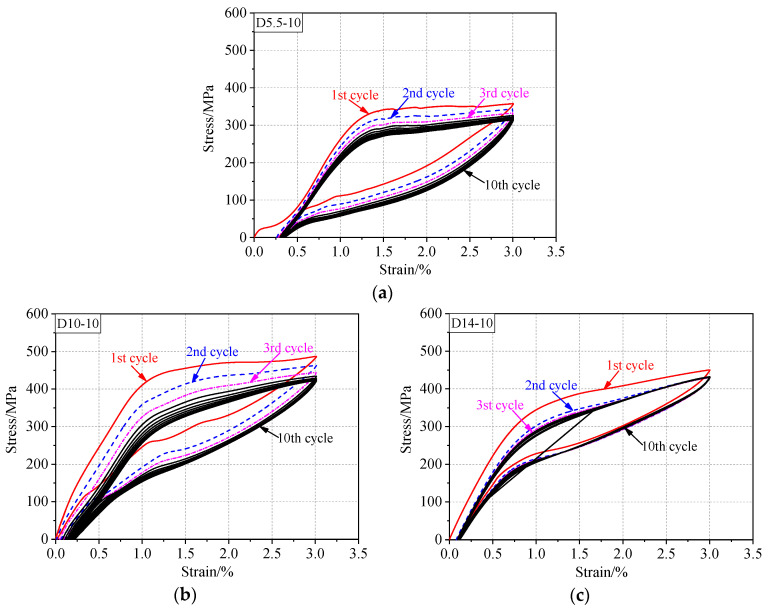
Hysteretic response under cyclic loading at constant strain: (**a**) hysteretic response of 5.5 mm, (**b**) 10 mm, and (**c**)14 mm.

Figure 8 shows the mechanical response of SMAs subjected to 10 cyclic loading–unloading cycles under the maximum strain of 3%. The mechanical response is of major concern because SMA components are typically subjected to cyclic loadings. Premature failures and significant degradation of their shape-recovery capabilities were observed during cyclic loadings. The loading plateau stress slightly decreased for successive cyclic loads (Figure 8). The most severe changes occurred in the first three cycles, and each cycle presented a decreasing incremental change from its previous cycle, thereby gradually approaching a limit cycle. This behavior is known as functional fatigue or shakedown [37]. Hence, the fatigue effect could be eliminated by “training” before use. Moreover, the residual strain progressively increased with cycling until stabilization was achieved. The stress–strain curve shows a smooth arc, and the hysteresis loop exhibited a shuttle shape with the increase in the number of cycles. Generally, after five cycles, the shape of the stress–strain hysteresis curve was roughly stable.

In addition, an obvious short plateau is observed in Figure 8a in the initial stage of loading during the first cycle. This phenomenon was mainly due to the R-phase transformation. R-phase is the intermediate phase between the austenite and martensite phases. An R-phase transformation with a small transformation strain (about 0.2%) was observed prior to the elastic response.

**Figure 9:**
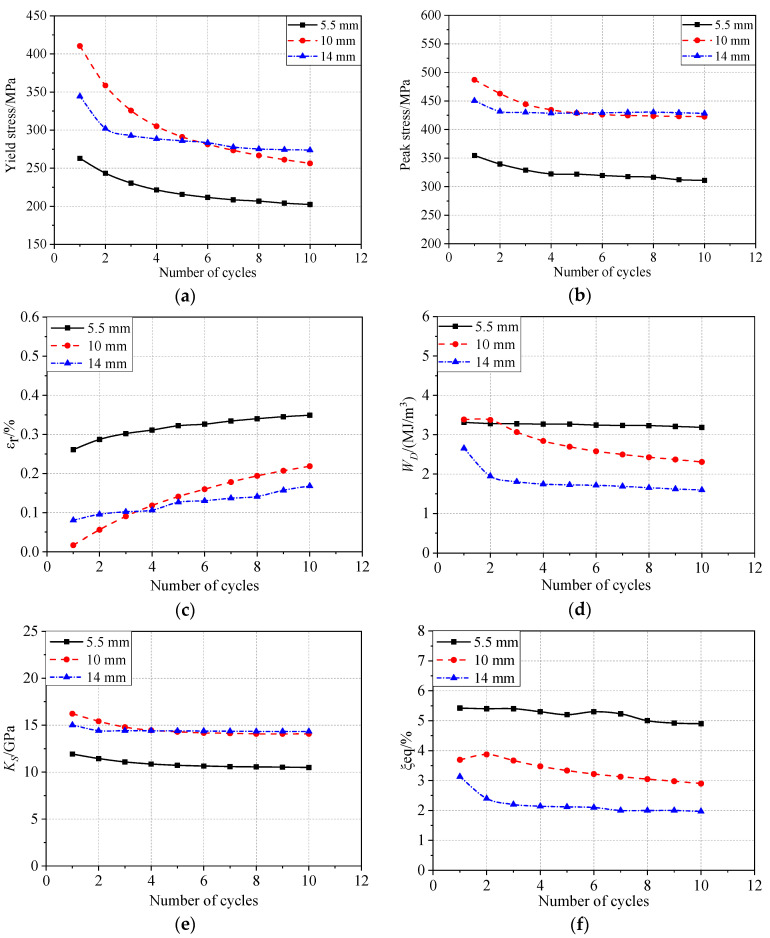
Comparison of the different mechanical properties for an increasing number of cycles: (**a**) yield stress, (**b**) peak stress, (**c**) residual strain, (**d**) energy consumption per cycle, (**e**) secant stiffness, and (**f**) equivalent viscous damping ratio.

Table 2 lists the values of the mechanical parameters in the first cyclic loading and the tenth cyclic loading. The yield stress and peak stress of the specimens with different diameters are shown in Figure 9a,b, respectively. The peak stress is the maximum stress obtained in cyclic tension. The most evident decrease in the yield stress and peak stress occurred during the first few cycles. The degradation of the yield stress seemed to be greater for the larger-diameter specimens; however, the results showed no apparent bar size effects. The residual strain increased with the number of cycles. After 10 cycles, the residual strain of the 5.5, 10, and 14 mm bars increased by 33.7%, 116%, and 110%, respectively, as compared with the values observed in the first cycle. However, the maximum residual strain was not more than 0.4%, and over 90% of the deformation was recovered. 

The evolution of the energy consumption, secant stiffness, and equivalent damping ratio under cyclic loading is described in Figure 9d–f, respectively, showing exponential-like decays in all cases approaching stable asymptotic values. For SMAs with different diameters, the larger the diameter, the smaller the area of the hysteresis loop (i.e., SMAs with small diameters easily show superelasticity and have a higher energy consumption capacity). However, the energy consumption and equivalent damping ratio decreased with increasing diameter. The equivalent damping ratio values showed distinct bar size effects (Figure 9f), which was attributed to a larger hysteresis loop for the smaller-diameter bar.

### 3.3. Increasing Strain Amplitude

**Figure 10:**
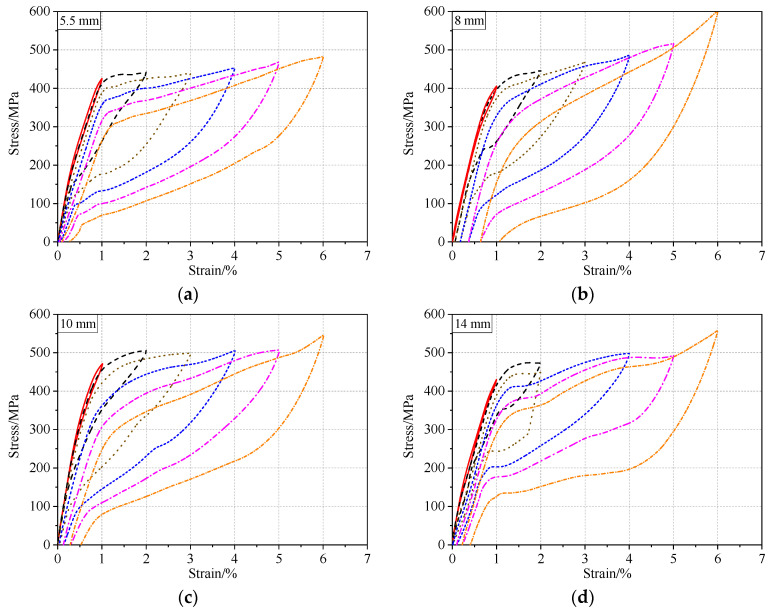
Hysteretic response under cyclic loading with increasing strain amplitudes: (**a**) 5.5 mm, (**b**) 8 mm, (**c**) 10 mm, and (**d**) 14 mm.

The majority of applications for SMAs in civil structures take advantage of their superelasticity to mitigate the response of structures during earthquakes. However, earthquake loads are often variable. Nevertheless, it is necessary to determine the effect of non-uniform cycles on the superelastic properties of SMA bars. Accordingly, the specimens were tested using a loading scheme of 1–6% strain cycles with increments of 1%. Figure 10 shows the hysteretic response of different-diameter SMA bars subjected to cyclic loading with incremental strain. These specimens were subjected to an annealing temperature of 400 ℃ for 15 min. One of the most prominent features of these charts is that the loading plateau stress of the specimens reduced slightly under the continuous cyclic loads. As shown in Figure 10, the 10 mm diameter sample presented a substantially higher strength than that of the other specimens by comparing the heights of the curves; this was mainly because of the differences in the material composition and some differences in the thermomechanical process. The 8 mm diameter bar showed the most obvious strain hardening and the highest stress at 6% strain.

**Figure 11:**
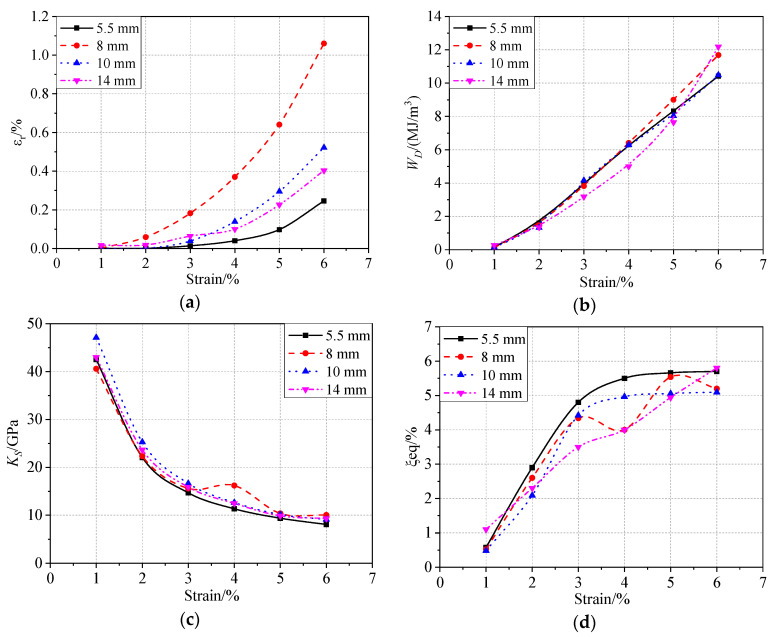
Comparison of mechanical behavior under increasing strain amplitudes: (**a**) residual strain, (**b**) energy consumption per cycle, (**c**) secant stiffness, and (**d**) equivalent viscous damping ratio.

Figure 11 shows a comparison of the mechanical characteristics under different strain amplitudes. The mechanical parameters of four diameters are summarized in Table 3, which lists the parameter values of the specimens subjected to 1% and 6% strain. The residual strain increased exponentially with increasing strain amplitude (Figure 11a). The highest residual strain was observed for the 8 mm bar; this was probably because of the low ambient temperature during the test. However, there seemed to be no relationship between the bar size and the residual strain. The secant stiffness $\kappa_{s}$ increased linearly before 3% strain, reached a maximum at approximately 4% or 5% strain, and then stabilized (Figure 11c). Additionally, the change trend of the equivalent damping ratio $\xi_{eq}$ was completely opposite to that of $\;\kappa_{s}$.

## 4. Conclusions

This study investigated the effects of the heat-treatment strategy, loading history, and bar size on the mechanical properties of SMA bars. It was determined that heat treatment greatly influenced the transformation stress of the SMA bars. On increasing the heat-treatment duration, the transformation stress clearly decreased. Generally, the heat-treatment strategy of 400 ℃ for 15 min was the optimal choice for the 14 mm diameter bars in order to obtain better transformation and recovery stress. Under cyclic loading at a constant strain of 3%, apparent degradations of the loading plateau stress were observed in the first three cycles, after which the hysteresis loops stabilized. The equivalent secant stiffness, equivalent damping ratio, and dissipated energy gradually decreased with an increasing number of cycles. With the incremental strain amplitude, the stress platform slightly reduced, and the mechanical characteristics changed significantly. These results could help in determining the appropriate use of SMAs in seismic applications. Presently, our team has utilized SMA bars in structural members, which has proved that superelastic SMAs can achieve the self-centering of a structure.

## Figures and Tables

**Table 1:** Specimens used in the test.

Set	Diameter (mm)	Heat Temperature (°C)	Duration (min)	Cycles
1	14.0	450	15	/
2	14.0	400	15/20/30	10
3	10.0	400	15	10
4	8.0	400	15	10
5	5.5	400	15	10

**Table 2:** Mechanical parameters of different specimens under cyclic loading.

Diameter (mm)	Yield Stress (MPa)		Peak Stress (MPa)		$\varepsilon_{r}\;(\%)$		$W_{D}\;(\mathrm{MJ}/m^{3})$		$K_{s}\;(\mathrm{GPa})$		$\xi_{eq}\;(\%)$	
	1st	10th	1st	10th	1st	10th	1st	10th	1st	10th	1st	10th
5.5	262.8	202.3	354.5	310.9	0.26	0.35	3.31	3.19	11.92	10.49	5.42	4.9
10.0	410.5	256.2	487.1	422.6	0.02	0.22	3.38	2.3	16.22	14.07	3.69	2.9
14.0	344.4	273.8	450.5	428.4	0.08	0.17	2.65	1.59	15.02	14.33	3.13	1.97

**Table 3:** Mechanical parameters of the different specimens under unequal cyclic loading.

Diameter (mm)	$\varepsilon_{r}\;(\%)$		$W_{D}\;(\mathrm{MJ}/m^{3})$		$K_{s}\;(\mathrm{GPa})$		$\xi_{eq}\;(\%)$	
	1%	6%	1%	6%	1%	6%	1%	6%
5.5	$5\times 10^{-4}$	0.25	0.156	10.42	42.52	8.06	0.58	5.7
8.0	$2\times 10^{-3}$	1.06	0.13	11.68	40.56	10.04	0.53	5.2
10.0	$7\times 10^{-3}$	0.52	0.14	10.49	47.11	9.10	0.48	5.09
14.0	0.017	0.41	0.28	12.18	43.01	9.30	1.1	5.8
